# Comparison of Diagnostic Value of Conventional Ultrasonography and Shear Wave Elastography in the Prediction of Thyroid Lesions Malignancy

**DOI:** 10.1371/journal.pone.0081532

**Published:** 2013-11-29

**Authors:** Ewelina Szczepanek-Parulska, Kosma Woliński, Adam Stangierski, Edyta Gurgul, Maciej Biczysko, Przemysław Majewski, Magdalena Rewaj-Łosyk, Marek Ruchała

**Affiliations:** 1 Department of Endocrinology, Metabolism and Internal Medicine, Karol Marcinkowski University of Medical Sciences, Poznan, Poland; 2 Department of General Surgery, Gastroenterological Oncological Surgery and Plastic Surgery, Karol Marcinkowski University of Medical Sciences, Poznan, Poland; 3 Department of Clinical Pathomorphology, Karol Marcinkowski University of Medical Sciences; Ospedale Pediatrico Bambino Gesu', Italy

## Abstract

**Introduction:**

Thyroid nodular disease (TND) is a very common disorder. However, since the rate of malignancy is reported to be 3-10%, only a minority of patients require aggressive surgical treatment. As a result, there is a need for diagnostic tools which would allow for a reliable differentiation between benign and malignant nodules. Although a number of conventional ultrasonographic (US) features are proved to be markers of malignancy, Shear Wave Elastography (SWE) is considered to be an improvement of conventional US. The aim of this study was to compare conventional US markers and SWE diagnostic values in the differentiation of benign and malignant thyroid nodules.

**Materials and Methods:**

All patients referred for thyroidectomy, irrespective of the indications, underwent a US thyroid examination prospectively. Patients with TND were included into the study. Results of the US and SWE examinations were compared with post-surgical histopathology.

**Results:**

One hundred and twenty two patients with 393 thyroid nodules were included into the study. Twenty two patients were diagnosed with cancer. SWE turned out to be a predictor of malignancy superior to any other conventional US markers (OR=54.5 using qualitative scales and 40.8 using quantitative data on maximal stiffness with a threshold of 50 kPa).

**Conclusions:**

Although most conventional US markers of malignancy prove to be significant, none of them are characterized by both high sensitivity and specificity. SWE seems to be an important step forward, allowing for a more reliable distinction of benign and malignant thyroid nodules. Our study, assessing SWE properties on the highest number of thyroid lesions at the time of publication, confirms the high diagnostic value of this technique. It also indicates that a quantitative evaluation of thyroid lesions is not superior to simpler qualitative methods.

## Introduction

Thyroid nodular disease (TND) is one of the most widespread endocrine disorders. While only about 3 - 7% of the population display palpable nodules, thyroid lesions in ultrasound (US) examination are reported in a large part of population. The exact prevalence differs strongly among studies, oscilating from about 10 to about 70% of the adult population or even more in women, the elderly or patients with certain particular conditions, such as acromegaly [1-4] Most studies estimate the risk of malignancy as quite low, within the range from less than 3 to about 10% [1,5,6]. These facts indicate a great need for diagnostic tools allowing a reliable distinction of nodules representing a high risk of malignancy. The decision whether to conduct surgery or follow-up is taken on the basis of thyroid US together with US-guided FNAB. Power Doppler (PD) examination and elastography are additional sonographic techniques, which are believed to increase the diagnostic value of conventional US [7].

Elastography is a sonographic method of assessing tissue stiffness. Numerous studies reported decreased elasticity of malignant thyroid nodules also in other conditions, e.g. chronic thyroiditis, Graves’ disease or subacute thyroiditis [8-11]. Shear Wave Elastography (SWE) is a new, promising, but still not widely available technique. It is thought to be more objective, reliable and reproducible than older variants of elastography, as it does not require any compressive maneuvers. In SWE, shear waves emission is induced by a focused ultrasonic beam. Based on the received signals, the elasticity of the tissue is assessed in real-time and may be estimated both qualitatively and quantitatively. In the case of the former, elasticity is expressed as a color. In the latter, elasticity of a particular region of interest is expressed in kPa, and is thought to be more objective than the previous qualitative method pattern [12,13]. Currently, the available reports on the usefulness of SWE are promising, but data are still limited due to the insufficient number of performed studies, as well as the number of evaluated patients. The present study is the first to comprehensively assess and compare the usefulness of the SWE using both a qualitative and quantitative method, as well as to compare the efficacy of this novel technique with traditional markers of malignancy detected in conventional ultrasonography on the largest number of nodules. 

The aim of this study is to estimate the diagnostic value of US, SWE and PD in the differentiation between benign and malignant thyroid tumors in a large group of patients undergoing thyroid surgery.

## Materials and Methods

### Patients

The Poznan University of Medical Sciences Ethical Committee approved this study and all participants provided informed written consent to participate in it. The study involved patients with diagnosed TND admitted for thyroidectomy between June and December 2010, irrespective of the indications for surgery. Finally, 122 patients met the abovementioned criteria and were enrolled in the study. 

### Conventional ultrasound, Shear Wave Elastography and Power Doppler examination

Conventional US, as well as PD and SWE, were performed using an AIXPLORER system by Supersonic Imagine and 2 - 10 MHz linear transducer. Examinations were performed before the surgery by four experienced sonographers (E.S-P., A.S., E.G., M.R). Elasticity of each thyroid nodule was assessed both qualitatively and quantitatively. A qualitative assessment of stiffness was performed with the use of 5-point Ueno and 3-point Rago scales [14,15]. According to the Ueno classification, lesions with grade I are entirely elastic as normal thyroid tissue, II – predominantly soft, containing areas of increased stiffness, III – elastic on the edges and rigid in the center, IV - present markedly increased stiffness in the whole nodule, and finally, entirely stiff lesions with stiff surroundings are classified as grade V. According to the Rago scale, pattern I is defined as a completely or predominantly soft lesion, III – as a completely or mostly stiff nodule, while II represents intermediate stiffness. For quantitative assessment of elasticity, two values of each nodule's stiffness expressed in kPa (maximal – Q-box max. and mean – Q-box mean) were recorded. 

Blood flow in PD was classified into five patterns which were defined as follows: I – absent blood flow; II - exclusively perinodular blood flow; III - perinodular and comparatively intense central blood flow; IV - central predominating over perinodular blood flow and V - exclusively central blood flow [16]. The following parameters were evaluated in conventional B-mode US: thyroid gland and lesion diameters, echogenicity (hypo-, hyper-, iso- and heterogenous), the presence of calcifications (micro-, macro- and egg-shell), shape (oval, round, “taller than wide”), margins (well defined or diffused), and structure (solid, predominantly solid, predominantly cystic, cystic).

### Histopathology

The final diagnosis of thyroid nodules was based on a histological examination performed after thyroidectomy by two pathologists as a routine medical procedure. 

### Statistical analysis

The calculations were performed using Statistica 10 from StatSoft. A P level of less than 0.05 was considered statistically significant. Odds ratios (OR), sensitivities and specificities were calculated for particular markers of malignancy. A P value below 0.05 was considered statistically significant. 

## Results

**Figure 1:**
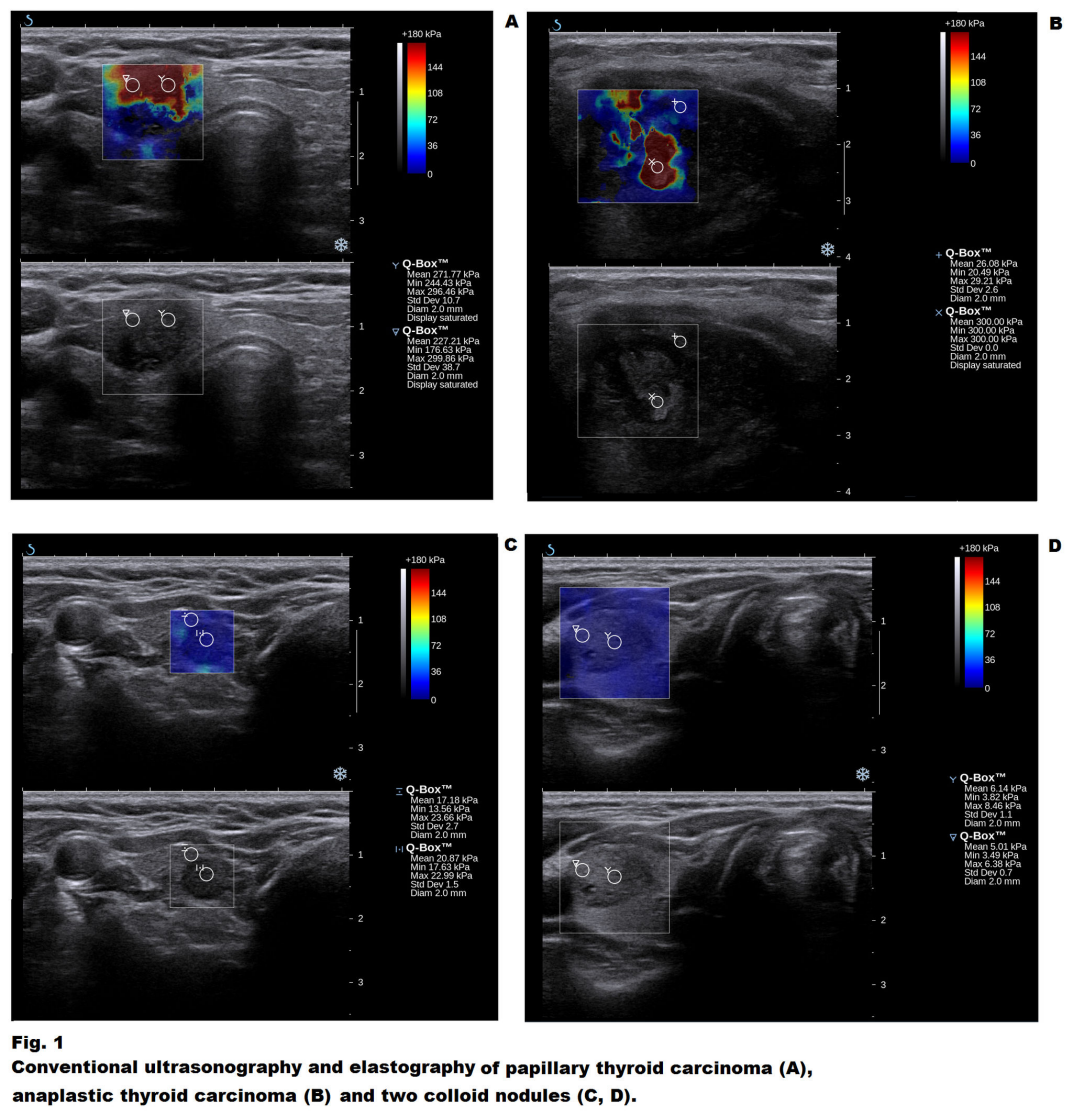
Conventional ultrasonography and elastography of papillary thyroid carcinoma (A), anaplastic thyroid carcinoma (B) and two colloid nodules (C, D).

One hundred and twenty two consecutive patients (103 men, 19 women), aged 23 to 78 years old (mean age – 51.0, standard deviation – 13.6, median – 52.0) with 393 thyroid nodules were included in the study. Twenty nine patients had solitary lesions, 93 – multinodular goiter (MNG). Twenty two nodules in 22 patients were histopathologically diagnosed as malignant. These cases included 18 papillary thyroid cancers (PTCs), two follicular thyroid cancers (FTCs), one medullary thyroid cancer (MTC) and one anaplastic thyroid cancer (ATC). Indication for surgery included: suspicion of TC in FNAB (16 patients), inconclusive results of FNAB (e.g. follicular tumor, repeated non-diagnostic biopsies) and/or suspicious sonographic picture in 42 patients, large nodular goiter (40 patients), coexisting nodular goiter and primary hyperparathyroidism (13 patients), toxic nodular goiter (8 patients) and nodular variant of Graves disease (3). Eighty five patients had FNAB prior to the surgery. Of the 22 cancers definitely diagnosed by histopathology, 15 were suspected in FNAB, two had inconclusive results, a further five patients with TC presented benign results of FNAB. All five overlooked TCs were PTCs. Conversely, among the 16 patients with suspicion of TC in FNAB, 15 cancer cases were confirmed by histopathology whereas one was not. US and SWE characteristics of all included nodules have been presented in table 1. Differences in mean and median stiffness between benign and malignant lesions have been presented in table 2, while some examples of the appearance of benign and malignant nodules in SWE have been shown in Figure 1. Table 3 demonstrates the usefulness of the two most commonly used qualitative scales (by Ueno and by Rago) in the differentiation of benign and malignant thyroid lesions.

**Table 1:** Sensitivity, specificity and OR of conventional US markers of malignancy, blood flow pattern on Power Doppler examination and elasticity assessed by Shear Wave Elastography.

**US pattern**	**Malignant**	**Benign**	**P**	**OR**	**Sensitivity %**	**Specificity %**
**Hypoechogenicity**	95.5%	65.9%	0.002	10.9 [1.4-81.7]	95.5 [77.2-99.9]	34.1 [29.4-39.1]
**Heterogenous echogenicity**	45.5%	56.1%	0.38			
**Microcalcifications**	42.9%	18.1%	0.001	3.4 [1.4-8.4]	42.9 [21.8-66.0]	81.9 [77.8-85.6]
**Macrocalcifications**	22.7%	7.8%	0.03	3.5 [1.2-10.2]	22.7 [7.8-45.4]	92.3 [89.1-94.7]
**Diffused Margins**	72.7%	26.4%	<0.0001	7.5 [2.8-19.6]	72.7 [49.8-89.3]	73.6 [69.0-78.0]
**Shape**						
Taller than wide	27.3%	7.5%	0.007	4.6 [1.7-12.7]	27.3 [10.7-50.3]	92.5 [89.4-94.9]
Oval	36.4%	54.5%	0.12			
Round	18.2%	28.4%	0.46			
**Composition**						
Solid	86.4%	59.2%	0.001	4.4 [1.3-15.0]	86.4 [65.1-97.1]	40.8 [35.8-45.9]
Predominantly solid	13.6%	33.9%				
Predominantly cystic	0%	9.8%				
**Solitary nodule [risk per nodule]**	31.8%	5.7%	0.0004	7.7 [2.9-20.9]	31.8 [13.9-54.8]	94.3 [91.5-96.4]
**Solitary nodule [risk per patient]**	31.8%	22.0%	0.45			
**Suspected lymph nodes**	9.1%	4.4%	0.32			
**Power Doppler**						
Pattern 3	30.8%	48.1%	0.26			
Pattern 4	30.8%	6.7%	0.01	6.2 [1.8-21.4]	30.8 [9.1-61.4]	93.3 [90.2-95.6]
Pattern 3 or 4	61.6%	54.8%	0.78			
**Elasticity (I – V)**						
≥2	95.5%	54.3%	<0.001	17.7 [2.4-133.0]	95.5 [77.2-99.9]	45.7 [40.7-50.9]
≥3	72.7%	15.5%	<0.001	14.5 [5.5-38.7]	72.7 [49.8-89.3]	84.5 [80.5-87.9]
≥4	59.1%	2.6%	<0.001	54.5 [18.9-156.7]	59.1 [36.3-79.3]	97.4 [95.3-98.8]
**Elasticity (I-III)**						
≥2	72.7%	15.5%	<0.001	14.5 [5.5-38.7]	72.7 [49.8-89.3]	84.5 [80.5-87.9]
≥3	59.1%	2.6%	<0.001	54.5 [18.9-156.7]	59.1 [36.3-79.3]	97.4 [95.3-98.8]
**Elasticity**						
Q-box max ≥50 kPa	95.2%	32.9%	<0.001	40.8 [5.4-307.8]	95.2 [76.2-99.9]	67.1 [62.0-71.9]
Q-box max ≥59 kPa	90.5%	27.8%	<0.001	24.7 [5.6-107.8]	90.5 [69.6-98.8]	72.2 [67.4-76.7]
Q-box max ≥65 kPa	81.0%	22.7%	<0.001	14.5 [4.7 - 44.1]	81.0 [58.1- 94.4]	77.3 [72.7- 81.4]
Q-box mean ≥49 kPa	85.7%	18.7%	<0.001	26.1 [7.5-90.4]	85.7 [63.7-97.0]	81.3 [76.9-85.1]
Q-box mean ≥42 kPa	90.5%	26.5%	<0.001	26.4 [6.0-115.4]	90.5 [69.6-98.8]	73.5 [68.7-78.0]
Q-box mean ≥38 kPa	95.2%	29.7%	<0.001	47.4 [6.3-357.6]	95.2 [76.2-99.9]	70.3 [65.5-74.9]

**Table 2:** Mean and median stiffness expressed in kPa in benign and malignant lesions.

	**Mean**	**SD**	**median**	**P**	**range**
**Q-box max [kPa]**					
Malignant	174.2	90.4	191.3	<0.0001	14.1-299.9
Benign	55.6	59.3	35.1		1.3-298.1
**Q-box mean [kPa]**					
Malignant	139.3	83.1	142.6	<0.0001	7.8-294.0
Benign	35.1	30.6	25.3		1.2-180.9

**Table 3:** Usefulness of two qualitative scales (by Ueno and by Rago) in assessment of benign and malignant thyroid lesions.

**Ueno scale**	**Malignant**	**Benign**	**OR**	**P**
I	4.55%	45.74%	0.06 [0.008-0.424]	0.005
II	22.73%	38.76%	0.46 [0.17-1.29]	0.14
III	13.64%	12.92%	1.06 [0.30-3.73]	0.92
IV or V	59.09%	2.58%	58.1 [19.8-170.6]	<0.0001
**Rago scale**				
I	27.27%	84.50%	0.07 [0.03-0.18]	<0.0001
II	13.64%	12.92%	1.06 [0.30-3.73]	0.92
III	59.09%	2.58%	58.1 [19.8-170.6]	<0.0001

## Discussion

The great prevalence of TND makes the distinction between benign and malignant lesions a vital problem in endocrinology. The introduction of sonoelastography in the assessment of thyroid lesions was hoped to be a milestone in non-invasive diagnostics of TND. Various techniques of tissue stiffness assessment gave promising results. However, the outcomes were strongly divergent. Giving the example referring to strain elastography, Trimboli et al. achieved an OR of 7.0, Asteria et al. – 68.9, while Gietka – Czernel et al. – 190.0 [17-19].

SWE is a novel technique, believed to be more reliable and reproducible than older variants of elastography. Previous studies on SWE demonstrated very significant differences in elasticity between benign and malignant lesions. In the study performed by Sebag et al. [9] OR of 89.1 was achieved using the threshold 65 kPa (with sensitivity 85.2 and specificity 93.9%); Veyrieres et al. [10] reported an OR of 37.9 (sensitivity 80.0%, specificity 90.5%) using 66 kPa as a cut-off point and reports high concordance between examinations performed by two sonographers. However, the amount of data concerning the usefulness of SWE in TND is still low. Particularly, there is very limited information on the results of using qualitative scales to assess SWE outcomes. 

This study confirms that SWE is indeed a valuable diagnostic technique, superior to any conventional US markers. Using quantitative data on maximal tissue stiffness, for a threshold equal to 65 kPa, adopted by Sebag et al. and similar to the one reported by Veyrieres et al. as optimal (66 kPa), we achieved comparable sensitivity and markedly lower specificity. Our results were close to those reported by Kim et al. [20]. According to this study, a threshold of 65 kPa yielded a sensitivity of 76.1% and a specificity of 64.1% (OR 5.7). In our study, the best OR was obtained for the cut-off point of 50 kPa, which was very sensitive and less specific. What is interesting, in our group the TC diagnosed preoperatively were found to present lower stiffness than those incidentally diagnosed in a histopathological examination. However, the studied group was too small to pursue a further interpretation of this finding.

Using the qualitative Ueno scale with a threshold of 4 points, or the Rago scale with a threshold of 3 points, the OR=58.1 was achieved with sensitivity slightly below 60%. Grade III in the Ueno scale is also a commonly used cut-off point improving sensitivity and decreases specificity. However, this grade itself is not a marker of malignancy (p=0.92), and is in fact equally common in benign nodules and TCs. Grade 1 can be considered as a marker of benignancy (OR=0.06, p=0.005), whereas, grade II was insignificantly more common in benignancies (p=0.14). According to our results, the use of quantitative data did not improve the diagnostic properties of SWE, as there was no cut-off point producing better OR than the qualitative scales. 

PD is another technique which is believed to improve the US diagnostic value. Patterns III and IV of blood flow are thought to be characteristic for malignancies. Our study confirms the significance of pattern 4 as a moderately strong cancer predictor (OR=6.2) and does not bear out the usefulness of pattern 3 (p=0.26).

Among conventional US markers, hypoechogenicity gained the highest OR (10.9), with sensitivity over 95% and poor specificity. In addition, the presence of diffused margins proved to be a valuable predictor of malignancy (OR=7.5) with sensitivity and specificity above 70%. According to our results, macrocalcifications the diagnostic significance of which is controversial [21,22], proved to be a significant malignancy predictor; however, one characterized by low sensitivity. A ”Taller than wide” shape was also more common in malignant lesions, while oval shape was only insignificantly more frequent in benign lesions. Our data suggest that solid composition is also a sensitive, but nonspecific risk factor (OR=4.4). Although 86.4% of TCs were completely solid, 13.6% of them were partially cystic. All of the cancers in our study was predominantly or purely cystic. Although solid nodules are believed to constitute a higher risk of malignancy, this issue remains a matter of debate. In the study performed by Bhatia et al. [23], all 19 TCs were solid, while 53% of benign nodules were partially cystic. According to Azizi et al.[20], solid nodules only insignificantly increased the risk of malignancy (73.3% *vs.* 66.0%). D’Souza et al. [22] report similar results (65.4% of cancers and 59.2% of benign nodules being solid – OR=1.3, 95% CI 0.5 - 3.1). However, unlike cancers, 10.3% of benign lesions were purely cystic.

Another controversial issue is the impact of nodularity on the risk of malignancy. Some studies have showed that solitary nodules present a higher risk [24], whereas others reported contradictory findings [25]. One possible reason for these discrepancies might be the fact that in some papers the risk was given per nodule [24], while in others per patient [25]. This difference can influence the outcome, especially when nodularity in the studied group is high, as it is in our research (on average 3.2 nodules per patient). According to our study, solitary thyroid nodules are at a greater risk of malignancy than nodules in MNG (p=0.0004, OR=7.7). Nevertheless, patients with solitary nodules are not at higher risk than patients with MNG (p=0.45). 

In conclusion, most conventional US markers of malignancy prove to be significant; yet, none of them ensure both high sensitivity and specificity. SWE seems to constitute an important step forward, allowing for a more reliable distinction between benign and malignant thyroid nodules. Our study, assessing SWE properties on the highest number of thyroid lesions at the time of publication, confirms the high diagnostic value of this technique. It also indicates that a quantitative evaluation of thyroid lesions is not superior to simpler qualitative methods.
